# Whole Genome Characterization of *Orthopoxvirus* (OPV) Abatino, a Zoonotic Virus Representing a Putative Novel Clade of Old World Orthopoxviruses

**DOI:** 10.3390/v10100546

**Published:** 2018-10-06

**Authors:** Cesare E. M. Gruber, Emanuela Giombini, Marina Selleri, Simon H. Tausch, Andreas Andrusch, Alona Tyshaieva, Giusy Cardeti, Raniero Lorenzetti, Lorenzo De Marco, Fabrizio Carletti, Andreas Nitsche, Maria R. Capobianchi, Giuseppe Ippolito, Gian Luca Autorino, Concetta Castilletti

**Affiliations:** 1 National Institute for Infectious Diseases Lazzaro Spallanzani IRCCS, via Portuense 292, 00149 Rome, Italy; cesare.gruber@inmi.it (C.E.M.G.); emanuela.giombini@inmi.it (E.G.); marina.selleri@inmi.it (M.S.); fabrizio.carletti@inmi.it (F.C.); giuseppe.ippolito@inmi.it (G.I.); concetta.castilletti@inmi.it (C.C.); 2 Robert Koch Institute, Centre for Biological Threats and Special Pathogens 1, Seestraße 10, 13353 Berlin, Germany; simon.tausch@bfr.bund.de (S.H.T.); andruscha@rki.de (A.A.); tyshaievaa@rki.de (A.T.); nitschea@rki.de (A.N.); 3 Istituto Zooprofilattico Sperimentale del Lazio e della Toscana M. Aleandri, via Appia Nuova 1411, 00178 Rome, Italy; giusy.cardeti@izslt.it (G.C.); raniero.lorenzetti@izslt.it (R.L.); gianluca.autorino@izslt.it (G.L.A.); 4 Parco Faunistico Piano dell’Abatino, via Capo Farfa 50, 02030 Poggio San Lorenzo, Italy; lorenzodem@gmail.com

**Keywords:** OPV Abatino, orthopoxvirus, cowpox virus, ectromelia virus, phylogenetic tree, core gene set

## Abstract

*Orthopoxviruses* (OPVs) are diffused over the complete Eurasian continent, but previously described strains are mostly from northern Europe, and few infections have been reported from Italy. Here we present the extended genomic characterization of OPV Abatino, a novel OPV isolated in Italy from an infected Tonkean macaque, with zoonotic potential. Phylogenetic analysis based on 102 conserved *OPV* genes (core gene set) showed that OPV Abatino is most closely related to the Ectromelia virus species (ECTV), although placed on a separate branch of the phylogenetic tree, bringing substantial support to the hypothesis that this strain may be part of a novel OPV clade. Extending the analysis to the entire set of genes (coding sequences, CDS) further substantiated this hypothesis. In fact the genome of OPV Abatino included more CDS than ECTV; most of the extra genes (mainly located in the terminal genome regions), showed the highest similarity with cowpox virus (CPXV); however vaccinia virus (VACV) and monkeypox virus (MPXV) were the closest OPV for certain CDS. These findings suggest that OPV Abatino could be the result of complex evolutionary events, diverging from any other previously described OPV, and may indicate that previously reported cases in Italy could represent the tip of the iceberg yet to be explored.

## 1. Introduction

The *Orthopoxvirus* (OPV) genus belongs to the *Poxviridae* family and includes viruses that have a typical brick-shaped morphology, and whose replication process lies in the cytoplasm of eukaryotic cells. The OPV genus comprehends a wide variety of zoonotic viruses that have vertebrates (mainly rodents) as their natural hosts [1]. Among OPVs, three species are recognized as endemic in North America and are referred to as new world OPVs: raccoonpox virus (RCNV), skunkpox virus (SKPV), and volepox virus (VPXV) [2], while seven species are recognized as originating from the eurasian continent, referred to as Old World OPVs: variola virus (VARV), vaccinia virus (VACV), camelpox virus (CMLV), monkeypox virus (MPXV), taterapox virus (TATV), ectromelia virus (ECTV) and cowpox virus (CPXV) [3].

Complete genomic sequences are now available for representative isolates of most OPV species. Many studies have underlined the importance of comparative genomics to analyze newly isolated OPV strains. Moreover, genome evolution and gene content analysis are widely used to investigate the genetic diversity of the OPV species, and to explore the association between genomic organization and phenotypic properties [4,5,6]. OPV genomes contain a single, linear dsDNA molecule, ranging in size from approximately 180,000 for VARV to more than 220,000 base pairs for CPXV. Two telomeres at the ends of the dsDNA genome form covalently closed hairpin structures at the termini. Near the termini, sequences responsible for the concatemer resolution of replication intermediates, as well as a variable series of direct tandem repeat sequences are located [7]. The inverted terminal repeats (ITR) lie at the ends of the viral genome and vary in size according to species. ITRs can contain coding regions for multiple genes, and genes contained within the ITRs can be present in multiple copies. For OPVs, the ITR size ranges between approximately 200 base pairs for VARV (which does not contain genes within its ITR), to almost 12,000 base pairs for VACV (which contains up to 6 diploid genes within its ITR). [8].

In the central region of the viral genome, syntenic gene locations and genome organization are generally maintained throughout the OPV species. The genes encoded in the central portion of the OPV genome are generally related to basic replicative processes, such as DNA replication, transcription, virion assembly and release. In OPVs, the central region accounts for around 75% of the genome and is the most conserved region within the genus [9]. On the contrary, genes encoded in the ends of the genome are more variable and, in some species, can be totally absent. These genes are usually involved in host interaction, pathogenicity and immunomodulation [10].

Most genetic and phenotypic diversity observed among OPVs is due to the contribution of heterologous recombination and duplication events, coupled with subsequent sequence divergence [4]. However, the progressive loss of functional viral genes through the accumulation of insertions and deletions, or other mechanisms that ultimately lead to gene truncations and loss was observed as an accompanying part of the process of host specialization and host range narrowing in OPVs such as VARV and ECTV [8]. Conversely, in parallel with the loss of genetic material, gene duplication or, more rarely, gene capture, could have contributed to the gain of different functions throughout time by several OPVs [11,12]. Phylogenetic reconstructions, based on sequence-derived inferences, reflect divergences in evolutionary history, while systematic analysis of variations in gene content can identify lineage-specific gene inactivation, deletions, duplications or recombination events. The combination of these methods can provide indirect information on virus host range and selective pressures [13]. The CPXV has the largest genome among the OPVs, with the highest number of genes hypothesized to be associated to host range [14]. The ECTV has a smaller genome and is associated to a narrow host range, with severe disease described in laboratory mousepox outbreaks [15].

In recent decades many OPV strains, mainly the CPXV, have been isolated from a variety of wild and domesticated animals. In humans and non-human primates, reports on CPXV infections are also increasing [16,17,18,19,20,21,22,23,24]. Recent findings based on whole genome sequencing describe the CPXV as being composed of multiple paraphyletic clades, although no association between CPXV clades and infected hosts was found [5,18].

OPVs are diffused over the entire Eurasian continent, but only a few reports describe OPV infections in Italy. One infection from an uncharacterized OPV was reported in a domestic cat from north-western Italy [25]; CPXV infections were observed in domestic ruminants from Sicily [26]; one CPXV infection in a llama was attributed to the introduction of an infected mouse by a German food distributor [27]. Our group analyzed the hemagglutinin (HA) and *crmB* gene sequences of two zoonotic infections in veterinarians from north-eastern Italy, who acquired the infection from cats in 2005 and 2007; almost identical OPV strains were involved in these cases, suggesting a possible segregation of these strains from other previously described OPV species [28]. A possibly novel OPV strain, isolated from a Tonkean macaque in 2015 in central Italy (OPV Abatino) has recently been described by our group [29]. The initial alignment of the whole genome for OPV Abatino indicated CPXV as the closest genome. Partial characterization performed on a restricted core gene set of 9 coding sequences (CDS) showed that this virus is in a distinct position with respect to all the OPV species and CPXV clades so far recognized [2,5,17,18,19]. Preliminary analyses also indicated OPV Abatino as mostly phylogenetically related to ECTV, suggesting that it could be part of a novel clade lying between the CPXV and the ECTV. This virus presumably also caused an asymptomatic infection in a worker involved in animal care at the same location [30]. In addition, another fatal OPV infection has recently been reported in Tuscany in a household cat [31], potentially representing a novel OPV clade, with high sequence homology with OPV Abatino within the restricted core gene set of 9 CDS.

In this paper we report the extended genomic characterization of OPV Abatino, focusing on a larger set (*n* = 102) of conserved CDS. In addition, a further comparative analysis with the most similar OPV genomes was based on the entire set of OPV Abatino CDS.

## 2. Materials and Methods

Whole genome sequencing was performed on the OPV Abatino isolate, obtained from a skin lesion of an affected monkey, using the metagenomic approach with the Ion Torrent next generation sequencing (NGS) PGM platform (Thermo Fisher Scientific Inc, Waltham, MA, USA), as described [29].

### 2.1. Whole Genome Assembly

The starting number of filtered reads obtained was 1,450,743. Target reads owing to the poxvirus genome (*n* = 157,852) were separated from raw reads using RAMBO-K software [32] with a k-mer length of 9 nucleotides. The resulting reads were de-novo assembled using both Velvet 3.6.2 [33] and SPAdes v. 3.11.1 [34] software. Major contigs were selected and aligned with BLAST [35], searching for the most homologous sequences, then aligned to homologous genomes with Geneious software v.1.8.0 [36], and merged in a unique contig. Bowtie2 v.2.2.6 [37] was also adopted for the confirmation of read separation by RAMBO-K, for coverage analysis and sequence control of the final contig.

### 2.2. Genome Annotation

The OPV scaffold was annotated using the PROKKA tool [38]. For a more accurate prediction, a set of annotated proteins were provided as an additional database, collecting all the CDS of three reference genomes: the ECTV Moscow (ECTV-Mos) strain, VACV Copenhagen (VACV-Cop) strain and CPXV Brighton-Red strain. ORF-prediction was performed with a minimum e-value of 10^−5^.

A second annotation procedure was also adopted using the VGA pipeline (unpublished), established at the Robert Koch Institute. The detailed description of this pipeline will be published elsewhere. Briefly, the VGA pipeline workflow is the following:

1. The pipeline takes the given genome, and searches for all ORFs longer than 93 bp (i.e. the size of the shortest annotated poxvirus gene).
2. Next, it translates ORFs into their corresponding amino acid sequences.
3. Then, it uses BLASTp to find orthologous genes for every ORF in a previously established database of all the protein sequences belonging to the family of *Poxviridae*.
4. Lastly, it collects the homologous genes of each ORF, together with their similarity values (see below), in a GenBank file.

All predicted CDS by either method were then merged and investigated using BLAST: all CDS identically predicted with both methods were maintained, all putative genes having low similarity with other OPVs were discarded and, when different putative CDS were predicted from the same genomic region, the ones most similar to database sequences were maintained. Finally, CDS were aligned to the assembled genome of OPV Abatino, correcting for the most reliable start and stop codons.

### 2.3. Core Gene Set Identification and Phylogenetic Analysis

Homologous CDS were selected using BLASTn, with a minimum e-value of 10^−5^, then manually checked; all CDS with a sequence length of <50% with respect to the mean length of the corresponding reference CDS were discarded. The core gene set was created collecting all the genes found in at least one representative strain for: CPXV, VACV, MPXV, CMLV, TATV, VARV, ECTV, and New World OPVs. The core genes were named according to VACV-Cop nomenclature, and their positions on the OPV Abatino genome were annotated.

The sequences of the core gene set were aligned with MUSCLE software v3.8.31 [39], and every gene alignment was manually controlled and corrected. The genes included in the core set were concatenated, giving rise to a total of 101,385 nt for OPV Abatino, and ranging between 99,957 and 101,514 for the 45 OPV genomes included in the analysis (Supplementary Table S1).

Phylogenetic analysis of the concatenated core gene set was performed with RAxML software 8.1.24 [40], using the GTRGAMMA substitution model and 10,000 bootstrap replicates.

### 2.4. Similarity Analysis

For each single homologous CDS, percent similarity scores were calculated, comparing OPV Abatino with the most phylogenetically related strains of CPXV (Ger2010-MKY strain) and ECTV (Moscow strain). The similarity values were computed using VGA software, translating each CDS into its amino acid sequence, aligning similar sequences, and dividing the number of identical positions in the amino acid alignment by the length of the query.

A comparison table was then produced including the CDS positions in the OPV Abatino genome along with the name of the VACV-Cop homologous gene, as well as the gene name, protein name and protein sequence similarity for the reference genomes (Supplementary Table S2). A similarity plot was then generated with every CDS of the annotated genomes along with their positions in base pairs on the annotated OPV Abatino genome. The CDS blocks are color coded, based on the level of their similarity to the homologous gene of either of the reference genomes (ECTV-Moscow or CPXV-Ger2010-MKY) using red and blue; orange was used where comparable similarity (i.e., with a ±0.01% tolerance in difference) to both reference genomes occurred (Figure 2). A BLASTn identity analysis, using default options, was performed on 23 CDS showing one of the following features: (i) no homologous CDS in both ECTV-Moscow and CPXV-Ger2010-MKY genomes; (ii) less than 90% similarity with respect of both reference genomes; (iii) no homologous CDS in one reference genome and less than 90% similarity with the other one. For this analysis, all OPV genomes included in the phylogenetic analysis were used for the database.

## 3. Results

### 3.1. Whole Genome Assembly

Three major contigs were obtained with de novo assembly. BLAST results found the CPXV MarLei07-1 strain to be the most similar genome (with an identity of 97% over 98% of the query cover). The three contigs were aligned with bowite2 on the complete genome of MarLei07-1 strain. Shorter contigs were then used to cover the gaps between the three major contigs. The consensus sequence of all contigs was than extracted to obtain the draft of the assembled genome of OPV Abatino. The ITR regions of the genome were not reconstructed and were excluded from genome assembly. All reads were than mapped to the obtained genome, and all variants were manually checked and corrected. The assembled whole genome of OPV Abatino (excluding ITR) resulted to be of 213,743 nt.

### 3.2. Genome Annotation

The number of putative OPV Abatino CDS recognized and manually corrected was 208. When possible, the VACV-Cop nomenclature was adopted for each CDS. The whole genome sequence, together with its annotation, is available in GenBank, with Accession Number: MH816996.

### 3.3. Core Gene Set Identification and Phylogenetic Analysis

**Figure 1:**
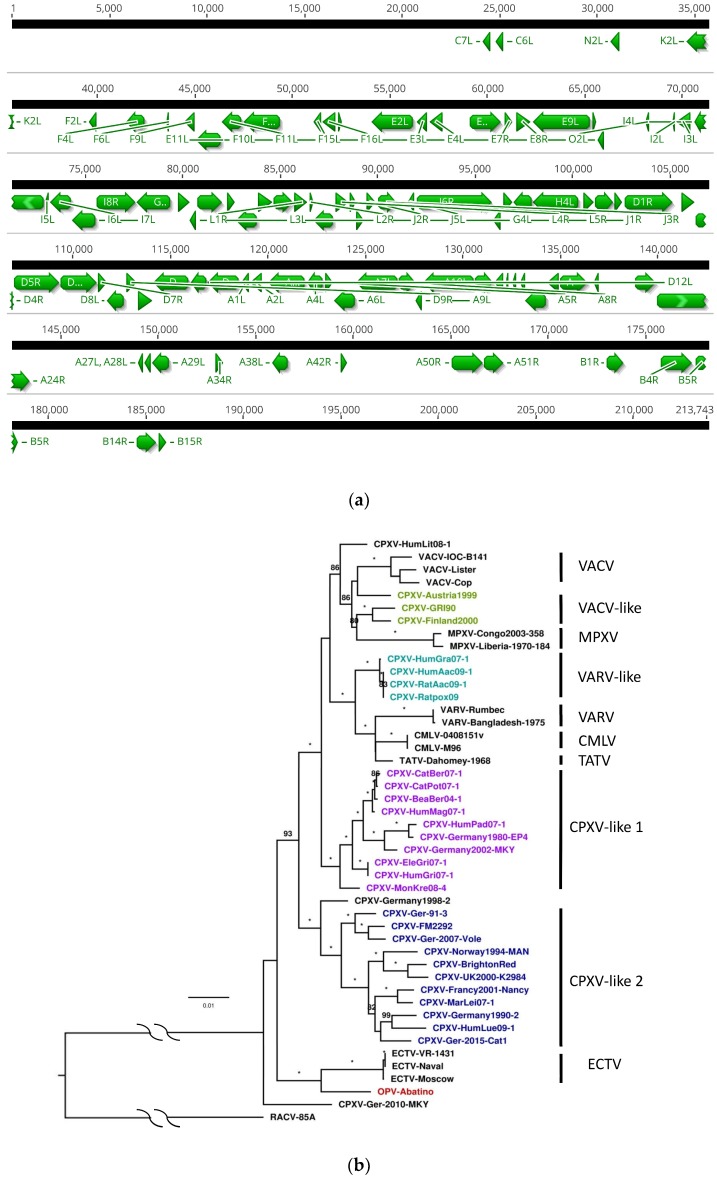
Location and phylogenetic analysis of the core genome of *Orthopoxvirus* (OPV) Abatino: (**a**) Localization of individual 102 coding sequences (CDS), highly conserved across all analyzed OPV, within the OPV Abatino genome. CDS are annotated following vaccinia virus-Copenhagen (VACV-Cop) nomenclature; (**b**) phylogenetic analysis of the core genome of OPV Abatino, built with the 102 concatenated CDS in the context of representative OPV genomes. CPXV clades, defined according to [2,5,17,18,19], are identified with color codes; three cowpox virus (CPXV) strains with discordant or no specified classification are also reported in black characters. The OPV Abatino strain is highlighted in red. A New World OPV, i.e. Raccoonpox Virus (RACV), was used as an outgroup. The CDS used in this analysis are detailed in Supplementary Table S1.

The core gene set was based on 102 CDS mainly located in the central part of OPV Abatino genome (Figure 1a), all included in the list of CDS described as conserved within Old World OPVs by Dabrowski et al. [17].

The phylogenetic tree of the 102 concatenated core gene set (Figure 1b) confirms the topology of the previous tree based on 9 conserved genes, with clustering OPV Abatino genome with ECTV, although placed on a distinct branch (bootstrap = 100) [29]. The tree also shows that the CPXV strain closest to OPV Abatino is a recently described strain, isolated from a non-human primate, belonging to a novel CPXV lineage (CPXV-Ger2010-MKY) [16].

### 3.4. Similarity Analysis

**Figure 2:**
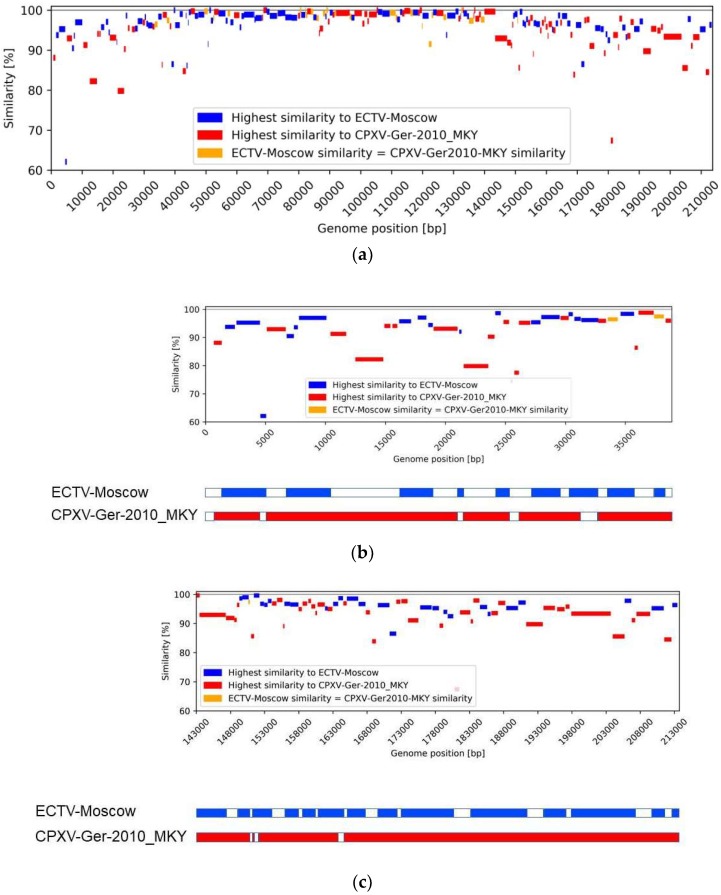
CDS similarity and gene synteny of OPV Abatino along their nt position, compared with the two most phylogenetically related OPV strains (Ectromelia virus-Moscow (ECTV-Mos) and CPXV-Ger2010-MKY). The CDS blocks are color coded: blue blocks represent the CDS with higher similarity to ECTV; red blocks represent CDS with higher similarity to CPXV; orange blocks represent CDS with comparable similarity to both reference genomes. (**a**) Similarity plot of the all CDS of OPV Abatino. (**b**) Similarity plot of left terminal region. (**c**) Similarity plot of right terminal region of the OPV Abatino genome. Bars below the plots show as empty boxes the regions of either ECTV-Mos or CPXV-Ger2010-MKY genome lacking homologous CDS in OPV Abatino.

Considering the complete CDS set of OPV Abatino (*n* = 208), the comparison with most phylogenetically related strains of CPXV (Ger2010-MKY strain) and ECTV (Moscow strain) is shown in Figure 2, where the Y axis corresponds to the highest similarity in percent. In particular, blue blocks represent those CDS with the highest similarity to ECTV; red blocks represent CDS with the highest similarity to CPXV; orange blocks represent CDS with comparable similarity to both reference genomes. The detailed similarity values of each CDS with the corresponding reference genomes is reported in Supplementary Table S2. As shown in Figure 2a, 47 of the 104 CDS located in the central part of the genome are blue, i.e., showed higher similarity (up to 100%) with ECTV; 33 CDS showed higher similarity with CPXV (red), and 24 CDS showed comparable similarity with both reference genomes (orange). In this part of the genome only one coding sequence of OPV Abatino (OPVA044 of Supplementary Table S2, not included in the core gene set) showed homologous CDS in CPXV (CPXV052, with similarity around 85%), but not in the ECTV genome. Moreover one CDS did not show homology in either CPXV or ECTV reference genomes (OPVA054).

A more complex pattern was observed in both terminal regions. These regions are known to be characterized by large insertions and deletions [8,12]. To better appreciate the similarity pattern and the syntenic gene locations in terminal regions, an enlarged visualization of these genome sections, including 104 CDS, is shown in Figure 2b and 2c. Similarity with the two reference strains is detailed in Supplementary Table S2. As can be seen, several OPV Abatino CDS located in the terminal regions show higher similarity with CPXV (*n* = 55), and many of them (*n* = 33) show homologous CDS only in the CPXV genome, while a few CDS are present only in the ECTV genome (*n* = 5: OPVA005, OPVA019, OPVA032, OPVA153, OPVA171) or are neither represented in the CPXV nor in the ECTV (*n* = 4: OPVA001, OPVA024, OPVA025, OPVA151).

Additional analysis was performed on 23 CDS (listed in Table 1) showing one of the following features: (i) no homologous (*n* = 5), (ii) low similarity (<90%, *n* = 6) with respect of both reference strains, (iii) no homologous CDS in one reference genome and less than 90% similarity with the other one (*n* = 12).

These 23 OPV Abatino CDS were compared by BLASTn to all OPVs used for the phylogenetic analysis, to identify the OPV genome with the highest identity. The results, shown in Table 1, indicate that the OPV genome with highest identity differed, depending on individual CDS, with identity range between 93.46% and 99.33%. In particular, 18 CDS showed their highest identity with various CPXV strains, and three showed highest identity with the VR-1431 strain of ECTV. For the remaining two genes, one showed highest identity with the VACV (Copenhagen strain) and one with the MPXV (Congo2003-358 strain) respectively; however, homologous CPXV genes were also found for both (see Supplementary Table S3).

## 4. Discussion

OPVs are currently considered zoonotic pathogens potentially re-emerging in Europe, paralleling the progressive annual decrease of individuals immunized against smallpox. In fact, an increased number of reports have recently described human OPV infections, exploring the variable pathogenic potential of different OPVs. A large genome confers to these viruses the ability to adapt to different hosts, providing increased opportunities for exploitation of humans or animals. Hence, extensive genetic characterization of emerging OPV zoonotic strains may help to elucidate the evolution mechanisms of the OPV, as well as their potential to expand their tropism to different animal hosts, and their potential as an epidemic threat for human populations. This might help to provide a valuable strategy for anticipating future disease threats.

Most of the described OPV isolates are from northern Europe, but large areas in western, southern, and eastern Europe are still not represented. Here we present the genome wide characterization of a novel zoonotic OPV recently isolated in Italy from a Tonkean macaque [29,30], based on the complete collection of its putative CDS.

We have combined full genome reconstruction, phylogenetic analysis and gene content variation analysis. To obtain the phylogenetic positioning of this novel Orthopoxviruses, all CDS homologous to any other previously described genomes, representative of OPV clades, were selected. In fact, as pointed out by Emerson et al. [41], more conserved genes are generally characterized by a relatively slower evolution, and they would not be influenced by gene loss.

The uniqueness of OPV Abatino depends on its phylogenetic proximity to ECTV, which is at a level higher than for any other OPV described before. This unusual characteristic is associated with a marked similarity of the central (most conserved) region of the genome, with many observed CDS more similar to ECTV than to any other OPV.

Non-conserved CDS of OPV Abatino were also analyzed, comparing them to the two most phylogenetically related OPV: CPXV-Ger2010-MKY and ECTV-Moscow.

The terminal regions of the genome seem more similar to this other OPV strain, described as CPXV. In particular, it is interesting to note that in those regions many putative genes, that are partially deleted or completely lacking in ECTV, are present in OPV-Abatino as well as in many CPXV strains. This suggests that, while the virus’ evolution may have been parallel to that of the ECTV, some characteristics of OPV Abatino (including the host range) may be similar to the CPXV. This is in agreement with the observation that OPV Abatino infected a group of Tonkean macaque, [29], and also caused a zoonotic infection as result of occupational exposure [30].

For the CDS for which no homologous counterpart was found, or that were highly divergent from the two most similar OPV genomes, we then searched for the OPV genome displaying the highest identity. The results indicated that the most similar genomes varied, including several CPXV strains, but also VACV and MPXV. This suggests that OPV Abatino could be the result of complex evolutionary events, diverging from any other previously described OPV.

For years, it has generally been supposed that, thanks to the mountainous territory, the occurrence of OPV infections in Italy could be limited to rare imported cases. Contrary to this idea, besides the zoonotic cases firstly reported in northern Italy [28], two events have recently been reported in central Italy. One event, described by our group, [29] was related to the virus here described, and caused also a probable zoonotic case [30]. Another OPV infection has recently been reported in Tuscany in a household cat [31]. These findings may suggest that the reported cases in Italy may represent just the tip of the iceberg yet to be explored.

## 5. Conclusions

The result of the present study, based on the sequence of 102 conserved OPV genes obtained by next generation sequencing, brings substantial support that this strain may be part of a novel, paraphyletic, OPV clade, with ECTV as the phylogenetically closest OPV species. The genome of OPV Abatino was larger than that of ECTV, including more CDS. Most of the extra genes showed their highest similarity with CPXV; however, for particular CDS, the closest OPVs were VACV and MPXV, suggesting that complex evolutionary events may be at the origin of this new virus. Future studies may elucidate the association between phylogenetic evolution of OPV Abatino and its host-range and pathogenic features.

New OPVs are being increasingly reported, most of them with zoonotic potential. The accurate genomic characterization of new OPVs may help to better define the molecular epidemiology and the evolutionary history of these viruses, and to get a better picture of their circulation in nature and the real challenges represented for animal and human health. In addition, detailed molecular characterization may help refining molecular diagnostic methods to be used in the management of human and animal infections.

## Figures and Tables

**Table 1:** OPV genomes showing the highest identity with OPV Abatino in the CDS showing no or low homology with the reference ECTV or CPXV. For the 23 CDS showing either no homologous (*n* = 5), or low similarity (<90%, *n* = 6) respect of both ECTV-Mos and CPXV-Ger2010-MKY reference strains, or no homologous CDS in one reference genome and less than 90% similarity with the other one (*n* = 12) a BLASTn identity analysis was performed with all OPVs included in the phylogenetic analysis (Figure 2). For each of the 23 CDS, the OPV genome with the highest identity (first hit in BLASTn analysis) is shown, together with its phylogenetic cluster.

OPV Abatino CDS	OPV Genome with Highest Identity in the Homologous CDS*	Phylogenetic Cluster **	% Identity with Homologous CDS in OPV Abatino
OPVA001	CPXV-Ratpox09	VARV-like	98.05
OPVA002	CPXV-HumLit08-1	***	99.09
OPVA005	CPXV-HumLue09-1	CPXV-like2	98.13
OPVA011	CPXV-GRI90	VACV-like	97.02
OPVA012	CPXV-GRI90	VACV-like	98.03
OPVA020	CPXV-Ratpox09	VARV-like	94.87
OPVA024	CPXV-Ratpox09	VARV-like	95.20
OPVA025	ECTV-VR-1431	ECTV	99.07
OPVA036	CPXV-HumLue09-1	CPXV-like2	97.00
OPVA040	CPXV-Germany1990-2	CPXV-like2	93.46
OPVA044	VACV-Cop	VACV	94.55
OPVA046	CPXV-MarLei07-1	CPXV-like2	95.79
OPVA054	ECTV-VR-1431	ECTV	99.33
OPVA151	ECTV-VR-1431	ECTV	99.22
OPVA152	CPXV-Ratpox09	VARV-like	94.85
OPVA159	CPXV-HumMag07-1	CPXV-like1	93.85
OPVA176	CPXV-Austria1999	VACV-like	97.15
OPVA178	CPXV-Germany1998-2	***	96.04
OPVA184	CPXV-Ger-2010-MKY	***	93.52
OPVA187	CPXV-GRI90	VARV-like	98.65
OPVA197	CPXV-Francy2001-Nancy	CPXV-like2	95.52
OPVA202	CPXV-GRI90	VACV-like	97.60
OPVA207	MPXV-Congo2003-358	MPXV	96.36

* first hit in BLASTn analysis; ** phylogenetic cluster of first hit, as described in Figure 1b; *** Genome not included in a cluster.

## Supplementary Materials

The following supplementary materials are available online at http://www.mdpi.com/1999-4915/10/10/546/s1: Table S1: OPV genomes included in this study. Table S2: Similarity values of each CDS of OPV Abatino with the corresponding reference genomes ECTV-Mos and CPXV-Ger2010-MKY. Table S3: Detailed data of 23 CDS of OPV Abatino showing one of the following features: (i) No homologous in both ECTV-Mos and CPXV-Ger2010-MKY reference strains (*n* = 5), (ii) Low similarity (<90%, *n* = 6) with respect of both reference strains, (iii) No homologous CDS in one reference genome and less than 90% similarity with the other one (*n* = 12).
